# Mothers Who Take Care of Children with Disabilities in Rural Areas of a Spanish Region

**DOI:** 10.3390/ijerph17082920

**Published:** 2020-04-23

**Authors:** Pablo A. Cantero-Garlito, Pedro Moruno-Miralles, Juan Antonio Flores-Martos

**Affiliations:** 1 Facultad de Ciencias de la Salud, Universidad de Castilla—La Mancha, 45600 Toledo, Spain; Pablo.Cantero@uclm.es; 2 Department of Philosophy, Anthropology, Sociology and Aesthetics, University of Castilla-La Mancha, 45600 Talavera de la Reina, Spain; JuanAntonio.Flores@uclm.es

**Keywords:** children, dependency, care, mothers, rural

## Abstract

The purpose of this research is to describe how the mothers of children with disabilities in rural areas of Extremadura perceive care tasks and the subjective impact that these tasks have on their daily life and health, as well as the subjective assessment that these mothers make of the benefits and services of the Spanish welfare policy. An interpretative paradigm was selected, using a qualitative approach and a phenomenological design. Twelve mothers were included. Data were collected through semi-structured interviews. A discourse analysis of the narrative information was performed using open, axial, and selective coding processes and the constant comparative method. Three topics have been extracted from the findings of the analysis: (1) extensive care responsibilities, (2) impacts upon well-being and daily life, and (3) resources that “barely” help. The care tasks performed by mothers of children with disabilities in rural areas have an enormous impact on their daily life and health. This involvement in caregiving generates a significant occupational imbalance which has an impact on their mental health, and which causes economic and social problems.

## 1. Introduction

Care tasks constitute an essential part of child rearing. These activities are especially significant for parents of any child, whose birth usually transforms their daily lives. However, these activities take on a special dimension when they are accompanied by chronic health problems or disability of the child [1]. In spite of the fact that there is a wide range of conditions which make it impossible generalize, many families will have to take over care which may extend throughout the child’s life, and which also implies a wide range of skills and the investment of much more time than that required to take care of a child who does not have a disability. These tasks can include support in basic activities such as feeding, dressing, hygiene, transport, communication, body mobilization, or play [2,3].

Childcare, and particularly care for children with disabilities, usually falls to women [2,3,4]. In many cases, the mother leaves her work to focus on the child’s care, and this has a major impact on her daily life, requiring a greater investment of time and resources [5]. This circumstance can increase stress and health problems in these women. In this regard, numerous studies have focused specifically on the stress experienced by mothers of children with disabilities [6,7,8,9,10,11,12], as well as the impact on their physical health [13] and the impact on occupational performance [2,3,14]. These health problems are more pronounced in mothers than in fathers of children with disabilities [15]. On the other hand, some authors have found that mothers of children with disabilities find enormous satisfaction and an extraordinary sense of vitality in caring for their children [13].

Additionally, some studies show that the mothers’ welfare is not significantly linked to the children’s health, but rather to economic and social factors [16]. Thus, children with disabilities are 3.5 times more likely to live with caregivers who experience financial difficulties and high levels of psychological stress than typically developing children [17]. When parents need to hold an ordinary job, in addition to caring for their children, the situation becomes precarious [3]. We should also bear in mind that the parents of children with disabilities are less likely to have a full-time job and suffer greater job insecurity, which leads to a lower income. Moreover, caring for a child with disabilities entails additional care-related expenses. In this regard, economic deprivation has proven to be an obstacle to resilience [18] and an additional source of stress for families of children with disabilities [19], situations which considerably increase perceived stress [20]. Regarding these aspects, different studies have highlighted that availability of financial support and of social and healthcare services increases caregivers’ quality of life [16].

Furthermore, when families of children with disabilities live in rural areas, in many instances, they have to face unique needs and additional barriers, compared to their urban counterparts, which are caused by geographical isolation [21]. Among other aspects, the specialized literature mentions transport difficulties to access rehabilitation services from rural areas, which means hours of travel, socioeconomic impact, and limited access to specialized medical services, social and healthcare resources and rehabilitation processes [1]. Rural areas present a significant deficiency in assistance and support services for mothers in order to provide the adequate care required by children, and there are limited options for the care of persons with disabilities and their families.

As Plaza points out [22], in Spain “the consequences of the demographic change show a considerable territorial inequality, which is particularly evident in rural areas whose situation has gradually worsened in recent times: hundreds of towns on the verge of depopulation, about two thousand villages empty and abandoned”. Thus, it is relevant to provide some data on the specificity of the study’s rural setting. This study was conducted in different counties of the province of Caceres, which belongs to the region of Extremadura, in western Spain. Here, the unemployment rate for women was 28.7% in the fourth quarter of 2019 according to the Labor Force Survey. The unemployment rate for rural women in Extremadura in 2018 (second quarter) was 28% in towns with less than 20,000 residents, according to Fundación FOESSA (Fomento de Estudios Sociales y Sociología Aplicada)—data from the VIII FOESSA Report and from the Survey on integration and social needs of Fundación FOESSA (EINSFOESSA) carried out in 2018 [23]. It is also worth stressing that the province of Cáceres lost five inhabitants per day, from July 2018 to July 2019, according to the Spanish National Statistics Institute (INE). Thus, Cáceres was among the six Spanish provinces with the greatest population decline for this period, when it experienced a decline of 0.52% of its total population.

Likewise, the three counties in northern Cáceres, which are the focus of our study, had a population loss of 14% (Granadilla and Valle del Ambroz) and 9.9% (Valle del Jerte) between 2000 and 2017—data from the project “Instituto Internacional de Investigación e Innovación del Envejecimiento” (4IE) (International Institute of Research and Innovation on Aging) from the Spanish National Statistics Institute and the Extremadura National Statistics Institute, provided by researchers from Universidad de Extremadura Dr. Lorenzo Mariano Juárez and Dr. Borja Rivero.

In order to address the care needs of dependent persons, the Spanish Government passed the Act 39/2006, of 14 December, on the Promotion of Personal Autonomy and Care of dependent persons (Dependency Act, from hereon). By December 2019, 1,395,037 people had been recognized as dependent under this Act in Spain. Among them, 66,618 people under the age of 18 (representing 5.92% of the total). The development of the Dependency Act in Spain has brought important benefits for dependent persons and their families: they have been granted the right for their needs to be met, economic benefits have been increased together with care resources (residences, day centers, home help services), and there has been a significant increase of jobs in the dependency care sector [24]. However, as some studies point out [25], the Dependency Act has proved to be “insufficient in practice due both to its design and to funding problems, which have revealed its limitations given the wide diversity and case mix of chronic diseases and have excluded a large number of persons who are not entitled to the subsidies or benefits provided by the Act”.

Nevertheless, there is very little literature on the experience of women who care for children with disabilities who are recipients of the Dependency Act, and even less from rural areas. Thus, the aim of this study is to describe how the mothers of children with disabilities from rural areas of Extremadura perceive the care tasks that they provide to their children and their subjective impact on their daily life and health, as well as the subjective assessment that these mothers make of the benefits and services of the Spanish welfare policy.

## 2. Materials and Methods

### 2.1. Study Design

To carry out this study, a qualitative approach based on discourse analysis and a phenomenological design were used [26,27]. As is well known, a phenomenological design focuses on the commonality of a lived experience within a particular group. Therefore, this allows for an improved description of how people understand and comprehend certain phenomena. The uniqueness of this perspective allows carry out a description of the essence of the experience for all of the participants; that is, this “what” they experienced and “how” they experienced it [28]. Thereby, we were able to explore, describe, and understand the meaning that participants gave to their reality and experiences.

### 2.2. Participants

The selection of participants was carried out between March and May 2019, following a theoretical purposive sampling. Inclusion criteria: (1) mothers of children with disabilities living in rural areas of northern Extremadura (Spain), that is, towns with less than 5000 residents and who would not have lived in cities during the child’s lifetime, (2) recipients of some type of benefit and/or service from the Sistema para la Autonomía y Atención a la Dependencia (System for the Autonomy and Care of Dependent Persons), the set of public and private services, as well as of economic benefits provided by the Spanish Dependency Act for the promotion of personal autonomy and care and protection of dependent persons and their families, and (3) those who voluntarily accepted to participate in the study by signing the informed consent form. Exclusion criteria: families whose children were 18 or older and/or receiving services not linked to the Dependency Act. The final sample included 12 participants (with an average age of 37.5). Their main sociodemographic characteristics are shown in Table 1.

The recruitment strategy was to contact different professionals from Non-Governmental Organization (NGO) of dependent persons from northern Extremadura -one of the Spanish regions with a larger number of rural towns- for a first selection among mothers whose children were users of the early care and rehabilitation program. The mothers were then contacted by telephone, in order to explain to them the aim of the study and to make an appointment at their convenience.

Once the meeting took place, the purpose of the interview was explained to them again and the information about the study was provided in writing. An informed consent form was also provided to be signed by all women participating in the study. This form stated that participation was voluntary, and that it would not have any direct implication for their children’s rehabilitation process but that it might be useful to improve care practices and professional interventions. It was reiterated that all information would be handled confidentially.

### 2.3. Data Collection

Two techniques were used to collect data for the field study: (1) an in-depth interview [27,29] guided by a series of semi-structured questions, although with the possibility of exploring and going into other topics which were not considered initially but arose during the interview [30]. (2) A fieldwork diary [31], which provides information about the context of the study and other elements to build a perspective of the participants’ lives. This can be useful when other investigators who did not attend the interview work with the information, and facilitates future analysis [32].

All the interviews were conducted between May 2019 and June 2019, in rooms of the children’s rehabilitation centers, while these had their rehabilitation sessions. These rooms met the appropriate conditions of privacy and comfort to have a dialogue where the mothers could express themselves sincerely.

The initial interview script was based on the objective of the study, following other related papers carried out in other settings or in other similar contexts (Table 2). The questions of the interview were subsequently modified to adapt them to the contents emerging from the participants’ accounts. These interviews were conducted by the same investigator, who has experience in qualitative research and in the use of in-depth interviews.

The data collection was carried out using audio recordings of the in-depth interviews with the mothers and the interviewer’s field notes. The field notes were collected during the different interviews and included data on the place of the interview, as well as observational information obtained from by participants before or after the interview. The duration of the in-depth interviews was between 90 and 120 min. The researchers agreed to finish the data collection once all the information was compiled and data saturation was reached.

To ensure anonymity, all the participants’ personal characteristics which could facilitate their identification were removed from this article. Once the interviews had been transcribed, the original audios were destroyed.

### 2.4. Data Analysis

The recorded interviews were subsequently transcribed and analyzed using the software ATLAS.ti 8.0 (Scientific Software Development GmbH, Berlin, Germany). The participants’ accounts were analyzed thematically. The different members of the research team met regularly for the duration of the study to analyze and review the data obtained. The participants’ accounts were initially coded by two of the investigators following an inductive approach, without pre-established analysis criteria or hypotheses.

The process of discourse analysis was carried out according to the follow sequence. Once the units of analysis were established by free-flowing data, an open coding process was carried out, following a constant comparison procedure [31,33] and a recursive analysis strategy [34]. Thus, 56 data codes were established, which were later grouped into 12 subcategories. Then, using an axial coding process [31], the subcategories were integrated into wider categories, based on a set of semantic linkers. Finally, the categories were grouped into three topics, corresponding to the study’s objectives, in which the participants’ experiences and significances are accurately grasped. The notes of the field diary were used during the process of coding and analysis of the accounts.

In order to increase and ensure the validity, trustworthiness and rigor of the research study [26,27,31], three triangulation strategies were followed: (a) methodological triangulation to obtain information (in-depth interviews and field diary); (b) researcher triangulation: in the data analysis process, the third researcher carried out a review analysis and external supervision of the analysis; (c) data source triangulation: different interviews were conducted with members of the NGO for persons with disabilities where participants were recruited and with health professionals, which enabled triangulation and contrasting of the information obtained. Finally, there was a return process of preliminary research results by means of a workshop with mothers of children with disabilities living in rural areas, which, from a participatory approach, allowed contrasting the findings of the study with the experience of other women in similar situations.

The consolidated criteria for reporting qualitative research (COREQ) guidelines [35] have been followed to ensure the quality of the study, and the 30 criteria of the checklist have been fulfilled.

### 2.5. Ethical Considerations

This study was approved by the Clinical Research Ethics Committee of the Integrated Health Management Area of Talavera de la Reina (Code 8/18). These research procedures were performed according to bioethical principles established in the Belmont Report, along with the Declaration of Helsinki and the Convention on Human Rights and Biomedicine of the European Council. All participants gave informed consent. Confidentiality was maintained throughout the study and only the research team has had access to the data obtained.

## 3. Results

The findings from the analysis of the interviews’ content have produced three categories which describe how mothers experience the day-to-day care of their children with disabilities and the impact it has on their daily life and health: (1) 24-h care; (2) Where do I get the time? Impact on daily life; and (3) Aids that “barely” help. The following explains each of these categories illustrated with quotes from the participants.

### 3.1. Extensive Care Responsibilities

For the women participating in this study, taking care of their children with disabilities is an activity that takes most of their time:

“…you have to feed him, dress him, take him to the toilet, you have to watch over him because, just like that, if he sees a staircase, he throws himself down. Or, if he sees a car in front, he stands there. So, you have to stay with him 24 h a day”

(M3).

The mothers’ daily routines and time organization are adjusted to those of their children with disabilities: school, home, transport to rehabilitation center, therapy, speech therapy, swimming pool, return to the village, home tasks, shower. As one of the participants say: “we are in a rush” (M2). During the time their children spend in the different rehabilitation sessions, the mothers stay in the waiting room, where they talk with other families (mainly mothers and grandmothers), creating an informal space of mutual help. Or else, sometimes, they take the opportunity to go shopping to nearby supermarkets or shops.

The fact that the rehabilitation sessions or attendance to other services usually take place in the afternoon conditions the children’s participation in leisure activities:

“This child, for example, on weekdays he does not have… He can’t go to the park, he can’t play with other children, he can’t, because he doesn’t have time in the afternoon”

(M3).

This sometimes conditions the schedule of activities of the other siblings: “Until now I took the sister with me, because I didn’t feel comfortable leaving her home alone” (M7). When the mother is not available, due to illness or other reasons, the children’s attendance to rehabilitation activities is affected:

“She was going to the swimming pool and also to the speech therapy, and when I fell ill, well she had to quit several things because, well, she couldn’t… She couldn’t, could she?”

(M2).

The mothers interviewed take the role of sole caregivers of their children with disabilities. In the case of married women, the fathers usually hold jobs with long working hours either in agriculture or outside the town where they live. Therefore, they do not participate in the caregiving routines, especially those related to body care (shower, dressing and feeding). Yet, fathers and siblings usually participate in activities such as walks, play both outdoors and at home, or occasional supervision. Therefore, these women find support for certain childcare tasks mainly from other women in the family. The role of grandmothers in these tasks is of enormous importance:

“I also had my sisters here, and they always, well, they have also given me a hand, right?: ‘Take the girl, pick her up for me’”

(M2).

“I have my mother, poor thing,… thank goodness she’s here, otherwise… Also on holidays, he goes with my mother. Because he can’t stay alone. Or with his sister”

(M3).

It should be especially stressed that most of the women have left their jobs in order to provide their children with the care they require in their daily lives. In other cases, these women have changed their work style and have looked for part-time jobs, working a few hours in the sector of home help for older persons. This allows them to keep a paid job for a few hours a day:

“I decided, well,… I was working when she was born. We had to give up something. Because this is too much, it’s a big burden. So, I don’t work and I’m completely dedicated to them”

(M4).

In addition, the mothers highlight that they also suffer problems heath due to environmental conditions related to rural areas:

“Here in town we’ve always had less things. We have always had to go to the city to shop, to go to the doctor, for everything… […] Women who do not have a university degree have only been able to work in agriculture or caring for the elderly. Always jobs that involve a lot of effort and little pay. That’s what there is…”

(M9).

### 3.2. Impact on Well-Being and Daily Life

Caregiving has direct implications on the mothers’ health. Thus, some tasks, like those which involve sudden movements or lifting heavy weights, have a negative impact on the mothers’ bodies and generate consequences such as back pain, insomnia, anemia, etc. One of the mothers, whose daughter uses a large wheelchair, points out:

“my arms are all messed up from pushing the chair up the slopes… and now she’s just a child… tomorrow”

(M7).

The impact on health is not only physical, but has also psychological consequences:

“It’s very hard mentally, not physically because physically people bear it up. And if you have strength, we bear up more, but mentally it’s very hard (…). It’s very hard because you see how other people can live their lives, you have limitations, no matter how much you want, you have limitations. People look ahead. You have to look at today”

(M11).

Moreover, they have a clear impact on a satisfactory and healthy occupational balance:

“I can’t go anywhere, I can’t go out on a Saturday, or a Sunday, or nothing at all, I don’t have a life”

(M3).

“You don’t take care of yourself, you can’t. Where do I get the time? I wish I could lose some weight. What nonsense, but I’m working from eight to three, I come, have lunch, I go and take a shower, I come here, I get home at half past seven. Do I take the girl and go for a walk? Do I have a Zumba lesson? No, I don’t, because on top of that I have to cook dinner, I have to do the laundry, I have to prepare the clothes for next day”

(M7).

Caring for a child with disability implies the mother’s giving up both work and other satisfying activities such as leisure activities. But especially, the daily involvement in care tasks makes mothers pay little attention to their own care, since care for others takes priority over self-care:

“Well, we’re young, we like to party, we also like going… going out at the weekend, go here, go there, and with him we can’t”

(M1).

“Me, nothing. I mean, the typical things: the girls, the house… of course you have to take care of yourself, but… When I can, because when I was having chemo I brought her with me, you know? I brought her and maybe I went to Carrefour, but… I had to sit down in any seat in Carrefour, I mean I was wiped out”

(M2).

However, despite giving up self-care and participation in satisfying activities, despite the impact on body and health, the mothers interviewed are satisfied with and proud of the care tasks for their children:

“This very often takes much effort, but you do whatever it takes for a child, don’t you? That’s what I think. And what shall I do, for myself? I don’t know… Yes, what I want is to be there for her. If I’m with her, well I’m at ease and I’m happy.”

(M2).

### 3.3. Resources that “Barely” Help

Among the range of resources, benefits and services offered by the Dependency Act in the region where these mothers live, all of them have opted to receive an economic benefit, therefore giving up other possibilities such as home help of third persons to take care of their children. The overall assessment of this economic aid is positive, but the mothers consider that it is insufficient to cover the cost of caring for a child with disability:

“it’s very good, but… it’s much too far, it’s not enough. With the issue of disability, they limit you very much: either you have money, a real fortune, or they limit you to home, school, or work those who have a job, therapy, and back home”

(M7).

Care of a child with disabilities entails a significant increase in a family’s expenses (medication, special diet, diapers, trips to the specialist, trips to the rehabilitation center, which is often many kilometers away from their town, in some cases they have to go seven days a week to a rehabilitation center that is more than 40 km away). As one of the participants says when she is asked what the aid is useful for:

“For nothing. Not even food. Because on top of that he has a special diet, he does not chew, everything has to be ground, he’s very allergic, everyday it’s special food for him, yogurts, his special juices. So as I say, it isn’t enough even for food, for nothing”, or as M7 complains: “For me, to pay the rent, not always to come here [Rehabilitation Center], because sometimes in winter, the butane bottles, the electricity, … (…) Who pays my rent that month? Fine, yes, I have a person who helps me to lift her. But from where if I don’t have a home?”

(M10).

Given the daily life needs of children with disabilities who live in rural areas, the economic aids from the public administration become a band-aid which support but do not transform the reality of families with a child with disabilities. Furthermore, these benefits have been reduced due to cutbacks after the economic crisis. This is largely due to the mothers’ work changes, whose income is reduced, and the costs of caring for a child with disabilities.

“And when the crisis began, they cut down dependency benefits. As if they were going to eat less. Or do less”

(M6).

Either families have resources or not, this barely covers basic expenses like transport, other supports, private professionals (speech therapy) or other services like swimming sessions. To the limited and reduced aids, we can add family situations in which there is only one stable wage in the case of married women, theirs being reduced if any, given their precarious jobs:

“I can’t go anywhere in the afternoons because I have to come here with him. He can’t quit his therapy because that’s what keeps him the way he is […] I do as much as I can and then it’s up to my mother. Well… there’s no other way. I can’t do more […] I can’t afford to fall ill, I can’t afford not to be able to go to work”

(M3).

In our opinion, in a rural setting with high levels of unemployment, the false dilemma presented by the Dependency Act becomes evident: either choosing to receive the help of a person a few hours a day, or choosing to be the caregiver who receives an economic aid for their children’s care. That “choice”, together with the option of a part-time job in home care of older or dependent persons, one of the few possible jobs in their towns and rural areas, would cause a care saturation and a greater impact on the health of mothers with both circumstances. One of the mothers explains it clearly:

“What I have is the money, because what do I want someone for to take care of him, if I am free, I’m unemployed?”

(M1).

## 4. Discussion

The women who take care of children with disabilities in rural areas who have participated in this study devote a significant number of hours every day to tasks related to their children’s care. The performance of these tasks has important implications on their daily lives and on their family environment. Many of these women have opted to give up work outside the family home or to choose part-time jobs mainly in the sector of home care services for older adults, in order to be able to dedicate more time to taking care of their children with disabilities, fact which is consistent with other studies [13,36]. As some authors point out [3] care of children with disabilities requires much more support than care of other children of the same age. Thus, many women have no other choice but to stop working in order to look after their children [37].

For many of them, this has meant to reorganize the family routines to adapt them to the needs and to the social, health and educational resources attended by their child. The children of the interviewed women attend different rehabilitation services, in line with some studies which indicate that, for example, the parents of children with autism use an average of four to seven therapies at the same time [38]. Participation in several therapies (such as speech and language, or occupational therapy) implies greater time demand, so parents spend much time both taking their children from their rural towns to the urban locations of rehabilitation centers [36], and waiting while their children receive their specialist sessions. All this results in less time to attend to other matters [39]. Likewise, even when the child is not present or they are sleeping, many of these mothers stay alert throughout the entire day or night [2,13,40]. Additionally, as Ranehov notes [3], the mothers try to get their children accepted by others, ensuring that society treats them with respect and dignity.

The women interviewed explain, to a greater or lesser extent, that care tasks have a significant impact on their health and well-being, which results in osteoarticular problems, sleep problems, obesity, etc. This is consistent with previous studies which show that caring for a child with disability is an important source of stress [10] and of occupational imbalance [41,42]. Caregiving leads many of the participants to neglect self-care and reduce the number of hours devoted to their own needs [36,43], and to activities linked to personal satisfaction and leisure [44], given the impossibility to participate in desired activities due to externally imposed demands.

Most participants face caregiving alone, since, if they have a male partner, he works outside the family home sometimes for long working hours, and therefore he only takes on more logistical care tasks and play [45] mainly on weekends. However, there is a strong commitment to care tasks among the immediate family circle, especially grandmothers, who become a source of family support in the form of cooperation in caregiving tasks, direct performance of these tasks, or economic support for the family environment. The literature has proven that grandmothers play a crucial role in the support of mothers and other family members, which is related to practical, economic, emotional, and affective support [46,47,48,49].

For some of the women who participate in this study, caregiving is a comforting task which gives them a meaning and provides them with social recognition from the community. There has been little research on these “positive” aspects of caregiving [13,50], but they constitute aspects of great interest given that the main focus of care tasks is usually placed on negative aspects, on their harmful effect on health, rather than on aspects of reaffirmation of identity and personal satisfaction.

Currently, there is a growing number of studies which suggest that environmental factors have a relevant role in parents’ well-being, especially socioeconomic and financial factors [16,17,51,52]. Caring for a child with disabilities in rural areas entails a considerable cost linked to the need of hygiene and personal care products which are not subsidized by the public administration, and of certain support products and in some cases prepared foods when there are intolerances or serious swallowing difficulties. Other costs should be added related to transport (gasoline, car repairs). In addition, as we have mentioned, in many of these families, the mother has had to give up their jobs or to find a part-time job, so the family income is sometimes low. Thus, in order to cope with these impoverished situations, some families opt for an economic aid to bridge certain gaps, instead of choosing to receive the help of a professional caregiver for their child. The use of economic benefits for dependency has been scarcely studied but a recent paper states that “the current socioeconomic context determines the economic benefit for dependency to act as an integration minimum income which allows meeting the basic subsistence needs of poor caregivers” [53].

Additionally, public debate has brought to the forefront in recent years a strong concern over the sharp demographic decline and abandonment of villages in rural areas, as well as the shortage and loss of public services, including transport and social and healthcare services, which are weakening the quality of life in those settings. The region of northern Extremadura where this study is located is clearly in this situation. Therefore, as Plaza points out, it will be necessary “to provide the population living there with the appropriate conditions to access basic services, giving priority to quality, because education, healthcare, or social services are inalienable rights, regardless of where one lives” [22].

In the same way, it is necessary to highlight how the rural context in which these children live with their mothers influences the construction of disability. In this sense, as reflected in different jobs [19,52,54], economic differences have a decisive influence on well-being. As reflected by the participants in this study, many of them live in a context that does not allow them access to well paid jobs. Although, as Resch [55] points out, access to resources and environmental and social supports have a greater effect on the well-being of parents than do the demographic variables of parents and children and the severity of the disability. To address these issues, Layton [56] suggests a series of recommendations that include attention to the subjective experience of environments and the application of human rights theory and the inclusive economy to address the multiple dimensions and levels of environments in working towards inclusion and well-being.

Within the limitations of this work, it is worth considering the exploratory nature of the study given the enormous difficulties in finding a sufficiently large sample of mothers with children with disabilities in the rural context under investigation. On the other hand, the sample was obtained among mothers of children with disabilities who attended a rehabilitation center in a nearby city. Thus, it has not taken into account family situations in which children usually stayed at the family home without attending assistance resources.

On the other hand, the mothers participating in this study have in common that they take care of children with disabilities in rural areas. However, the children have different disabilities and are of different ages.

As regards future lines of research, it should be noted that many of the studies on women’s care tasks focus on the negative aspects of these tasks for the daily life, health and well-being of caregivers. It would be interesting to take a close look at aspects related to satisfaction and personal recognition, sense of purpose, creation of a role socially valued in rural areas. It would also be worth going deeper into the experience of men providing care in rural contexts, as well as the implications of caregiving for their daily lives and the well-being of their children with disabilities.

## 5. Conclusions

In conclusion, our study evaluates the perspectives of mothers of children with disabilities who live in rural areas. It becomes clear that the mothers’ occupational routines are adapted to the needs of children with disabilities, which implies a very significant daily time commitment. The care tasks are performed fundamentally by mothers, with occasional support from other women in the family, while fathers carry out logistical tasks, play, etc. This creates a significant occupational imbalance in women, which has an impact on their somatic, social, and mental health and causes economic and social problems. We would like to highlight that the impact of environmental factors on mothers’ health is one of the most important issues related with caregiving. Evidently, the environmental conditions of rural areas affect also directly the mother’s health. They suffer similar problems as their children with respect to accessing to health services.

The children who live in rural settings do not have equal access to healthcare services as children from urban areas. Additionally, the aids that they receive from the administration hardly enable the adaptation and transformation of family environments, which may imply a more equitable access to continuous quality care. Finally, the mothers are constantly concerned about their children’s future, especially when they are no longer able to perform care tasks or when they die.

Based on the findings of this study, caregiving in rural areas should involve interventions which address the needs of the families of children with disabilities, especially their mothers who take charge of caregiving. It is very important to make appropriate care possible in the environments where the children live, where their peers are, implementing programs which allow families to become involved in an inclusive manner: advice, assessment, and provision of mothers’ health needs, support to families, home care, professional teams who go to their areas, as well as approaching community-based models of care in local contexts.

## Figures and Tables

**Table 1:** Characteristics of participants.

Participant	Age	Marital Status	Children	Age (and Sex) of the Child with Disability	Level of Disability
M1	47	Married	2 children	17 (boy)	Serious
M2	45	Married	2 children	17 (girl)	Serious
M3	44	Separated	3 children	8 (boy)	Moderated
M4	38	Married	2 children	12 (girl)	Very Serious
M5	44	Married	1 child	10 (boy)	Serious
M6	35	Married	2 children	11 (boy)	Very Serious
M7	37	Single	1 child	9 (girl)	Very Serious
M8	38	Married	1 child	6 (girl)	Serious
M9	37	Married	2 children	2 (boy)	Very Serious
M10	43	Married	2 children	2 (boy)	Very Serious
M11	40	Married (second marriage)	2 children	14 (girl)	Moderated
M12	30	Married	1 child	3 (girl)	Serious

**Table 2:** Interview script.

Interview Script
- What caregiving tasks do you perform?
- How much time do you spend on those tasks?
- Who else cooperates in the caregiving tasks? What do they do?
- How do you think your child’s care has changed your life?
- How has your child’s care had an impact on your health?
- How do you take care of yourself?
- What support have you found from professional resources? What do you think of the support received? How do you think more help could be offered?
- What does caring for your child mean for you?
